# People act when they feel obliged. Prosocial intentions toward Ukrainian refugees in Poland during the first two weeks of the war in Ukraine

**DOI:** 10.5114/cipp/177007

**Published:** 2024-01-16

**Authors:** Iwona Nowakowska

**Affiliations:** Institute of Psychology, The Maria Grzegorzewska University, Warsaw, Poland

**Keywords:** moral foundations, personal responsibility, prosocial intentions, refugees, war in Ukraine

## Abstract

**BACKGROUND:**

In February 2022, Poland became one of the most engaged countries in accepting Ukrainian refugees. Based on the norm activation model, the study, performed during the first two weeks of the war, examined the prosocial intentions of Poles toward Ukrainians in relation to the individualizing moral foundations (harm/care and fairness/reciprocity), as well as beliefs about the obligation of individual citizens to help.

**PARTICIPANTS AND PROCEDURE:**

The study was designed to investigate the views of Poles on helping Ukrainians in times of tension. On the second day of data collection, the war in Ukraine began. Therefore, the study shows the intentions of Poles to help refugees from Ukraine in the first days of full-scale war. A total of 139 people aged 18-71 years from the general public participated (102 females) in an online survey distributed through social media channels.

**RESULTS:**

The results show that individual obligation belief fully mediates the effect of individualizing moral foundations on prosocial behavior intentions.

**CONCLUSIONS:**

Based on the results, it could be concluded that during a refugee crisis, in line with the norm activation model, highlighting the personal obligation to provide support can be important to motivate people to help others in need. The effect of a sense of personal obligation is more important than the effect of an underlying individualizing morality.

## BACKGROUND

The outbreak of the war in Ukraine on February 24th, 2022, led to an unprecedented wave of war refugees coming to Poland (Chrzan-Dętkoś & Murawska, 2023). In the first months of the war, Poles, as citizens of the country hosting refugees, donated resources to satisfy the immediate needs of the refugees (Kyliushyk & Jastrzebowska, 2023) and engaged in volunteering to provide more long-term help (Domaradzki et al., 2022). These grassroots initiatives among Poles were crucial then, as the available systemic solutions were scarce (Ociepa-Kicińska & Gorzałczyńska-Koczkodaj, 2022). Longitudinal studies have shown that Poles showed more positive attitudes toward Ukrainians in 2022, following the outbreak of the war, compared to 2021 (Babińska et al., 2022).

According to the contact hypothesis (Allport, 1954), such contacts may result in greater acceptance for the representatives of a group of others (compared to groups without such contacts). Ukrainians before the war were often economic migrants in Poland, which increased the chances for mutual contact between these two nations (Jaroszewicz, 2018). Ukrainians are deemed quite close to Poles (or, more broadly, Europeans) in their culture (Nowak et al., 2023). It has also been observed since the outbreak of the Russian war, as Poles have shown greater acceptance towards Ukrainian refugees than refugees from other countries (Babińska et al., 2022). Data from December 2022 showed that as many as 8 million war refugees from Ukraine crossed the Polish border, and 1 million stayed there (Duszczyk et al., 2023). It can be assumed that a serious armed conflict in a culturally close and relatively well-known neighboring country may be perceived as a threat to Poles and cause social support mobilization to help Ukrainian citizens who fled the war zone and those who stayed in Ukraine (Isański et al., 2022).

Although social support is a behavior observed at the level of groups, it relies on individuals’ efforts (Uehara, 1990). The norm activation model is one of the well-verified models of prosocial behaviors concentrating on individuals (Schwartz, 1977). It has been tested in a variety of prosocial contexts. Recently, it has been found to be relevant for predicting civic engagement (Rosenthal & Yu, 2022), charitable shopping (Borusiak & Kucharska, 2022), and compliance with COVID-19 mitigation strategies (Rui et al., 2022).

According to this theory, feelings of moral obligation to perform a specific act are based on considering the values and norms to which the act relates. The stronger a person finds a particular norm for self-evaluation, the more profound is the feeling of moral obligation to behave in a particular way. Schwartz (1977) proposed the following process leading to an eventual prosocial action: first, people perceive the need of others – become aware of the situation, notice actions they could do, recognize their abilities, and accept responsibility for an eventual act. Next, the normative structure (values, norms) is activated, generating a feeling of moral obligation. Typically it takes place unconsciously. In the subsequent step, people weigh the costs and consequences of an action and reevaluate the situation to respond with action or inaction.

The process can also be observed in the prosocial intentions toward Ukrainian refugees. The information in the media regarding the approaching wave of refugees and the potential needs created the initial perception of a need to help. Moreover, such a phenomenon could have occurred for a prolonged period of time due to armed conflicts in Ukraine taking place since 2014 (Prykhodko, 2022). Depending on the moral values essential to people, the feeling of moral obligation could have been formed, resulting in a willingness to engage in help or not.

It is worthwhile to integrate the view of the norm activation theory with the moral foundations theory (Graham et al., 2009). Moral foundations theory is based on insights from evolutionary and anthropological sciences (Zapko-Willmes et al., 2021). The theory proposes five central moral foundations: harm/care, fairness/reciprocity (grouped into an individualizing or person-focused morality), authority/respect, ingroup/loyalty, and purity/sanctity (grouped into a binding or group-focused morality; Graham et al., 2009, 2011; Pagliaro et al., 2021). Similarly to the moral considerations described in the norm activation model, moral foundations are based on the assumption that people judge morality-related issues without conscious effort (Graham et al., 2011). Moreover, moral foundations have been researched in the case of political contexts, and it has been found that political liberals endorse the individualizing morality, whereas political conservatives endorse the binding morality (Day et al., 2014; Graham et al., 2009).

What moral values could be especially relevant in the refugee acceptance context? In the case of an armed conflict, people who witness the suffering of others may feel compassion (Zaki, 2020) but also feel that what happens is not fair or that the violence does not make sense (James, 1995). The individualizing moral foundations seem especially relevant to this context. Individualizing morality highlights the importance of avoiding harm, caring for the well-being of individuals, and protecting civic freedoms (Graham et al., 2011; Haidt & Joseph, 2008). For people endorsing this type of morality, society should be organized to promote inclusion (Kugler et al., 2014). Individualizing morality might result in compassion for refugees, who are typically described using harm/care-related language (Hoewe et al., 2022). Moreover, war and its injustice may be viewed as a specific form of lack of cooperation (as cooperation opposes aggression associated with war) and trigger anger towards those considered aggressors, which may further empower compassion towards those considered victims. Thus, endorsement of individualizing moral foundations can generally translate into a more prominent feeling of solidarity with war victims and, consequently, prosocial behaviors such as charitable giving.

Individualizing morality relates positively to charitable giving (volunteering and donations) to the outgroup members (Nilsson et al., 2020). It is also related to self-transcendence values from the basic human values theory (Schwartz et al., 2012; Zapko-Willmes et al., 2021). These values encourage people to go beyond their interests to protect other individuals’ well-being (Schwartz, 1992) and are typically activated in the case of prosocial behaviors (Caprara et al., 2012; Schwartz, 2010). Individualizing moral foundations are also related to agreeableness (Lewis & Bates, 2011), a predictor of prosocial behaviors stemming from emotional reactions to witnessing someone needing help (Habashi et al., 2016).

However, as mentioned above, according to the norm activation model (Schwartz, 1977), sole moral norms may not be enough for prosocial behaviors. The person has to accept responsibility for their actions, and the internal feeling of obligation should activate the norms to translate into intentions and eventual behaviors.

In the case of the Ukrainian war refugee crisis, a person from the receiving country (Poland) can endorse individualizing morality highly, but feels that it is not the obligation of an individual citizen to help refugees. Instead, they might consider that authorities or specialized nongovernmental organizations should prepare and implement support. Only when a person feels bound to act can the norm be active. However, moral norms can be linked to moral obligation belief, as they need to exist before forming it (Schwartz, 1977). First, a person has a system of moral values they endorse. Next, in response to the perceived need of others, they activate them and feel obliged to act. The relationship between moral norms and prosocial intentions can thus be indirect. That is why it can be hypothesized that:

H1. The feeling of the moral obligation of individuals to help Ukrainian refugees is a mediator of the relationship between individualizing moral foundations and prosocial intentions.

This hypothesis will be tested based on data gathered in the first days of the war in Ukraine, and prosocial intentions will encompass the willingness to perform four behaviors toward the Ukrainian refugees: donate resources, donate money one time, donate money regularly, and volunteer. Additionally, age, gender and socioeconomic status will be controlled as they could relate to prosocial intentions (see a review by Bekkers & Wiepking, 2011). Binding moral foundations will also be controlled given their potential relation to negative behavioral intentions toward an outgroup (Hadarics & Kende, 2018).

## PARTICIPANTS AND PROCEDURE

### PARTICIPANTS

A total of 139 people aged 18-72 (*M* = 27.06, *SD* = 9.21) took part in the study, 102 females (73.4%), 30 males (21.6%), and seven people who preferred not to declare gender (5.0%). Eleven people lived in a village (7.9%), 17 in a town with up to 50,000 inhabitants (12.2%), 13 in a town with 50,000-100,000 inhabitants (9.4%), 35 in a town with 100,000-500,000 inhabitants (25.2%) and 63 in a town with over 500,000 inhabitants (45.3%). Regarding the last stage of education finished, two people (1.4%) had primary school education, 2 (1.4%) vocational education, 60 (43.2%) high school, 43 (30.9%) BA, 28 (20.1%) MA, 3 (2.2%) a PhD or a higher academic degree, and 1 (0.7%) reported having other education. For socioeconomic status, on a scale of 1-10 (1 – *I cannot afford basic expenses*, 10 – *I can afford everything that is necessary and spare money*), for this sample *M* = 7.01, *SD* = 2.20. Two people (1.4%) reported having Ukrainian family roots. Eighty-four people (60.4%) reported having at least one form of contact with Ukrainians (having Ukrainian friends, neighbors, or other regular contacts with Ukrainians).

### PROCEDURE

The study was designed to show the social beliefs regarding helping Ukrainians as the public discourse in Poland has begun to address the topic of the potential refugee wave coming from this country due to political instability. The data collection began on February 23, 2022, and on the following day, the invasion of Ukraine began. To capture the moment of the strictest social mobilization, the data were purposefully collected for the two weeks of the war and finished on March 9, 2022. The participants were recruited on social media – groups for students, city inhabitants, groups designed for research data collection, and Visible Hand groups. Respondents were informed about the purpose of the study as “finding out opinions about supporting Ukrainians in Poland”. All participants provided informed consent and were not remunerated for taking part in the survey. There were no consequences for quitting the survey at any time. The study was anonymous, and stopping filling out the survey before completion did not result in recording a partial response. The research was approved by the Maria Grzegorzewska University Research Ethics Committee (decision no. 75/2022).

### MEASURES

*Moral foundations* were assessed with the Moral Foundations Questionnaire (MFQ; Graham et al., 2011; Polish version: Jarmakowski-Kostrzanowski & Jarmakowska-Kostrzanowska, 2016). It is a self-report consisting of 30 items in which people describe how important to them are the foundational moral domains in moral decision-making: harm/care, fairness/reciprocity (which constitute the individualizing morality), ingroup/loyalty, authority/respect, and purity/sanctity (which constitute the binding morality; Graham et al., 2009; Pagliaro et al., 2021). The scale, in general, consists of two parts. In the first one, people decide how important it is for them to consider an issue when making a moral decision, for example: “Whether or not someone acted unfairly”. In the second one, people decide whether they agree with the provided moral statements; for example: “Compassion for those who are suffering is the most crucial virtue”. The participants answer on a 6-point Likert scale from 1 (*not at all relevant*) to 6 (*extremely relevant*). The reliability of the individualizing moral foundations index in the current study was α = .74, and that of the binding moral foundations index was α = .90.

*Individual obligation belief* was assessed with a statement of our own construction: “I think that an obligation to help Ukrainians relies mostly on every Polish citizen”. The participants answered from 0 (*totally disagree*) to 100 (*totally agree*).

*Prosocial intentions toward Ukrainian refugees* were assessed with a survey of our own construction. The participants answered four questions regarding the intention to donate resources other than money to a/the Ukrainian refugee/s, intention to donate money for one time to a/the Ukrainian refugee/s, intention to donate money regularly to a/the Ukrainian refugee/s, and volunteer for a/the Ukrainian refugee/s. The participants answered from 0 (*totally disagree*) to 100 (*totally agree*). The index of prosocial intentions was computed as a mean of four items. Cronbach’s α for the scale was .89.

### SOFTWARE

All analyses were performed using SPSS 28.0.1.0 for Windows. The PROCESS 4.0 macro for SPSS was used to test moderation and mediation (Hayes, 2018). Assumption check was assisted by the Heteroskedasticity V3 macro for SPSS (Daryanto, 2020).

## RESULTS

### NOTE ON DATA

Raw data are publicly available at https://osf.io/eh87c/.

### DESCRIPTIVE STATISTICS AND CORRELATION ANALYSIS

Descriptive statistics and correlations between study variables are presented in Table 1.

**Table 1:** Descriptive statistics and correlations between study variables

Variable	*M*	*SD*	1	2	3	4	5	6	7
1. Individualizing moral foundations	4.99	0.52	–						
2. Individual obligation belief	59.22	34.91	.24^**^	–					
3. Prosocial behavior intentions	65.65	29.70	.28^**^	.69^***^	–				
4. Age	27.06	9.21	.09	–.02	–.04	–			
5. Gender (0 – female, 1 – male)	–	–	–.42^***^	–.13	–.18^*^	.00	–		
6. Socioeconomic status	7.01	2.20	–.01	–.01	–.01	.06	.05	–	
7. Binding moral foundations	3.17	0.84	.05	–.15	–.16	.11	.13	.09	–

*Note*. ^*^p < .05, ^**^p < .01, ^***^p < .001.

Data from Table 1 suggest significant and weak correlations between individualizing moral foundations, individual obligation belief, and prosocial intentions toward Ukrainian refugees, and a moderate correlation between individual obligation belief and prosocial intentions toward Ukrainian refugees. It should be noted that the average results indicated relatively high prosocial intentions (*M* = 65.65, for a scale of 0-100). Out of the controlled variables, only female gender correlated positively with prosocial intentions and individualizing moral foundations. That is why only gender was taken into account as a covariate in the subsequent mediation analysis.

### MEDIATION ANALYSIS

**Figure 1:**
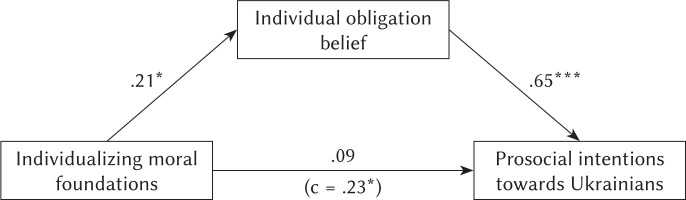
Results of the mediation analysis *Note*. ^*^p < .01, ^***^p < .001.

Next, we performed a mediation analysis with bootstrapping for *N* = 1000, predicting prosocial behavior intentions toward Ukrainians with individualizing moral foundations as the dependent variable and individual obligation belief as a mediator, with gender as a covariate. The results of the analysis are provided in Table 2 and visualized in Figure 1.

The mediation analysis suggested that individualizing moral foundations were related to individual obligation belief, and individual obligation belief was related to prosocial behavior intentions. Prior to entering the mediator into the model, individualizing moral foundations predicted prosocial behavior intentions. However, individual obligation belief fully mediated the relationship between individualizing moral foundations and prosocial behavior intentions, and after entering the mediator in the model it was found to insignificant. Gender was not related to any of the variables in the path.

**Table 2:** Testing the mediation effect of individual obligation belief in the relationship between individualizing moral foundations and prosocial intentions toward Ukrainians (gender controlled)


Dependent variable: Individual obligation belief, *F*(2, 129) = 3.58, *p* < .05							
	*B* [95% CI]		SE	*t*	*p*	*R* ^2^	
Gender	–.04 [–.23; .15]		.10	–0.46	.645	.05	
Individualizing moral foundations	.21 [.02; .40]		.09	2.19	.030		
Dependent variable: Prosocial intentions toward Ukrainians, *F*(3, 128) = 39.51, *p* < .001							
	*B* [95% CI]		SE	*t*	*p*		*R* ^2^
Gender	–.06 [–.20; .08]		.07	–0.83	.410		.48
Individualizing moral foundations	.09 [–.05; .23]		.07	1.27	.206		
Individual obligation belief	.65 [.53; .78]		.07	10.00	< .001		
Indirect effects on prosocial intentions toward Ukrainians (gender controlled)							
		Effect [95% CI]	Boot SE				
Individualizing moral foundations → Individual obligation belief → Prosocial intentions toward Ukrainians		.14 [.02; .27]	.06				
Total indirect effect of Individualizing moral foundations on Prosocial intentions toward Ukrainians		.23 [.04; .41]	.09				

## DISCUSSION

The purpose of this study was to test the potential mediating role of belief in an individual obligation to help in the relationship between individualizing moral foundations and prosocial behavior intentions towards Ukrainian refugees in Poland in the very first phase (two first weeks) of the conflict in Ukraine.

The analysis fully supported H1. It suggested that individual obligation belief fully mediates individualizing moral foundations and prosocial behavior intentions toward Ukrainian refugees. Moral obligation needs to stem from a moral rule – in this case, the individualizing foundations that underline other people’s welfare. The broader landscape of Ukrainian refugees’ depiction in the Polish media during the war must also be considered here. It was found that Ukrainians were described as “war refugees” and discussed mainly in terms of how they cope and are received in Poland (Zawadzka-Paluektau, 2023), strongly suggesting their victim status and positively affecting attitudes toward them. Furthermore, the cultural closeness of Poles and Ukrainians (Nowak et al., 2023) or the issue of identifying oneself and Ukrainians as members of the European community (Politi et al., 2023) could play a role in higher empathizing with their needs and resulted in a sense of responsibility for their well-being in Poland.

However, without underlying morality concerns, there would be no obligation to fulfill. Moreover, staying congruent with one’s own morality (due to complying with the feeling of moral obligation) lets one preserve a good self-image (Schwartz, 1977). The immediate effect of giving can be feeling good about oneself and being convinced that one is building a good reputation, acting according to moral norms (Bekkers & Wiepking, 2011; Jasielska & Rajchert, 2020). It may also relieve the feeling of guilt about others being in a worse situation (Nowakowska, 2022) (which may be true as the recipients were war victims), especially considering that people of high individualizing moral foundations value fairness/reciprocity that underlines justice and equity (Graham et al., 2009). Giving is also a form of prosocial behavior that can maintain justice and equity in interpersonal relations (Krebs, 1982), presumably in intergroup relations between war victims and the members of the country accepting refugees.

In conclusion, the study has provided general support for the norm activation model (Schwartz, 1977) in its mediation variant. It must be acknowledged that the model was tested in the context of helping outgroup members in the face of an armed conflict. However, it needs to be considered that this is specific to one nationality’s members and that the aspect of Polish-Ukrainian relationships cannot be ignored here, potentially influencing how these relationships between variables were shaped.

### LIMITATIONS AND FUTURE RESEARCH DIRECTIONS

The study was correlational and cross-sectional, capturing only the relationships between variables and not the causal relationships. It is also based on self-report of intentions, which prevents us from finding out the actual behaviors towards Ukrainian refugees. The timing of the study was specific, which is a strength of the study, as it shows a unique period of vast importance; however, it does not enable us to make statements about how the propensity to support Ukrainian refugees was shaped throughout the war. The study was also based on one Polish population, which has historical connections with Ukraine that, on the one hand, may promote care and friendly contact (e.g., a long period in the newest history in which Ukrainians were migrating to Poland to work), but on the other hand may promote hostility (e.g., World War II events and conflicts between Polish and Ukrainian communities in the territories of contemporary Western Ukraine). The sample was also relatively small and derived from convenience sampling, which compromises representativeness and only allows medium-sized statistical effects to be shown. Finally, the study by design consisted of variables related to individual differences in morality and consideration of consequences. It did not consider the broader social context of Polish-Ukrainian relations or the quality of previous personal experiences (own or family’s) with Ukrainians. Future studies should be carried out with different methodologies (e.g., longitudinal studies, diary studies, qualitative studies: interviews with Poles and with refugees) to identify patterns of social support mobilization in Poland in response to the conflict in Ukraine and to prevent hostile attitudes towards the refugees entering the country.
